# Patients’ motivation for undergoing elective repair of a primary ventral hernia: a Danish nationwide cohort study

**DOI:** 10.1007/s10029-026-03627-5

**Published:** 2026-03-06

**Authors:** Anders Gram-Hanssen, Jason Joe Baker, Hugin Reistrup, Jacob Rosenberg

**Affiliations:** Center for Perioperative Optimization, Department of Surgery, Herlev Hospital, Copenhagen University Hospital, Borgmester Ib Juuls Vej 1, Herlev, DK-2730 Denmark

**Keywords:** Ventral hernia, Patient motivation, Mixed-methods, AFTERHERNIA, Elective surgery

## Abstract

**Purpose:**

This study aimed to explore patient motivations for undergoing elective repair of a primary ventral hernia and to estimate the proportion of patients who underwent surgery for indications that are not traditionally prioritized in surgical decision-making.

**Methods:**

This descriptive mixed-methods study was based on a nationwide survey of Danish patients who had undergone elective repair of an umbilical or epigastric hernia between 2014 and 2024. Data were collected through structured survey questions on predefined motivational factors and linked to national clinical registries. Qualitative free-text responses were analyzed using systematic text condensation to provide contextual understanding of patient motivations. The study forms part of the AFTERHERNIA Project, a series of nationwide studies investigating patient-reported outcomes after groin and ventral hernia surgery in Denmark.

**Results:**

A total of 18,753 participants completed the survey, corresponding to a response rate of 82%. The most frequently reported motivations for surgery were pain or discomfort (63%), concerns about hernia growth (39%), and doctors’ recommendations (34%). 12% of participants selected only reasons classified as motivations that are not traditionally prioritized in surgical decision-making. (cosmetic concerns, fear of growth, fear of incarceration, or emotional impact). The qualitative analysis supported these findings, identifying seven themes that reflected symptom burden, professional guidance, and contextual or practical factors as key drivers of patient decision-making.

**Conclusion:**

About one in eight patients underwent elective repair of a primary ventral hernia motivated by indications that are not traditionally prioritized in surgical decision-making. However, most respondents were motivated by physical symptoms or professional advice.

## Introduction

Primary ventral hernias, including umbilical and epigastric hernias, are common conditions that may cause pain, discomfort, or functional limitations, which can significantly impact patients’ quality of life [1]. The only definitive treatment for primary ventral hernias is surgical repair, and it is preferably performed in an elective setting [1]. Surgeons’ decision-making in this regard is primarily guided by evidence-based treatment guidelines, which are informed mainly by studies focused on recurrence rates and surgical complications [1–3]. However, the patients’ decision-making process before undergoing elective hernia repair may be influenced by a wide range of patient-centered factors, such as symptom severity, concerns about hernia progression, and the impact on daily activities or psychological well-being [4]. Understanding these diverse motivations is important for optimizing patient care and informing shared decision-making between patients and surgeons. Previous studies have highlighted that patient-reported outcomes are key in evaluating the success of hernia surgery beyond traditional clinical metrics [5–8]. However, the specific reasons why patients choose to proceed with elective surgery remain understudied. Given the wide spectrum of hernia-related symptoms, ranging from minimal discomfort to significant impairment, a more detailed exploration of patients’ motivations for seeking surgical intervention is needed.

This study explores patient motivations for undergoing elective repair of a primary ventral hernia using a descriptive mixed-methods approach. Using a combination of quantitative survey data and qualitative text analysis, this study aims to provide a comprehensive understanding of the factors driving patients to opt for surgery. Furthermore, the study will estimate the proportion of patients who pursue surgery for indications that are not traditionally prioritized in surgical decision-making.

## Methods

This is a descriptive mixed-methods study based on a nationwide survey of participants residing in Denmark with lived experience of elective primary ventral hernia surgery [9]. The study includes a quantitative component based on structured survey responses from participants, and a qualitative component involving systematic text condensation of participants’ written responses to open-ended questions. A mixed-methods design was chosen to complement quantitative data on predefined motivational factors with qualitative insights from patients’ own words, thereby providing a more comprehensive understanding of why individuals opt for elective ventral hernia repair. The study is supported by data linkage with clinical and intraoperative data from several nationwide databases. The study was reported according to the Good Reporting of a Mixed Methods Study (GRAMMS) guideline [10]. The present study is part of the AFTERHERNIA Project [9], which is a series of nationwide studies assessing the long-term clinical and patient-reported outcomes of groin and ventral hernia surgery in Denmark.

### Participants

In the AFTERHERNIA Project, participants were identified using surgical procedure codes indicating a ventral hernia repair in the Danish National Patient Registry and recruited digitally through the Danish Digital Post service, a personal communication service provided by the Danish government to all Danish residents [11]. Patients were included if they had undergone a ventral hernia repair in Denmark between 1 January 2014 and 31 March 2024 and were ≥ 18 years of age at the time of surgery. In this study, we only included patients who had undergone an elective repair of a primary ventral hernia, including both umbilical and epigastric hernias. Exclusion criteria included: death, emigration, exemption from using digital communication with the government, inability to complete the questionnaire due to cognitive or physical impairment, or insufficient knowledge of Danish.

### Survey

All participants received an electronic survey in Danish inquiring about their motivation for undergoing surgery. The survey also included questions about demographics and surgical outcomes. The survey was distributed to all eligible participants in Denmark on one of three separate days: 24 August 2024, 13 October 2024, or 9 November 2024. Each potential participant received one invitation letter followed by three automated reminders. The questionnaire included a series of closed-ended questions with structured response options, as well as an open-ended question with a free-text response option. The lead-in question of the survey was: “Before your operation, what were the main issues that motivated you to have a hernia repair?”. It was possible to select multiple pre-defined response options simultaneously. Four of the response options were classified as motivations not traditionally prioritized in surgical decision-making regarding primary ventral hernias: cosmetic concerns, concerns about the hernia enlarging, concerns about incarceration, and mental/emotional impact.

### Registry data

The survey responses were linked to demographic, clinical, and operative data from the Danish National Patient Registry [12], the Danish Ventral Hernia Database [13], and the Danish Civil Registration System [14] using a mandatory personal identification number assigned to all residents of Denmark.

### Qualitative analysis

A qualitative analysis was performed using systematic text condensation to identify, analyze, and interpret patterns of meaning within the free-text data provided by participants. The free-text data were processed and filtered, and entries without analytical value, such as “OK” were excluded before data analysis. The intended goal of the qualitative analysis was to uncover potentially overlooked aspects of patient motivation not covered by the prespecified types of motivation.

Systematic text condensation entails four analytic steps [15]:

1. Total impression – from chaos to themes: gaining an overall understanding of the data.
2. Identifying and sorting meaning units – from themes to codes: identifying segments of text containing information relevant to patients’ motivation for surgery.
3. Condensation – from code to meaning: summarizing the core content across similar meaning units.
4. Synthesizing – from condensation to descriptions and concepts: developing coherent thematic descriptions grounded in the data.

The initial total impression was established by reading all free-text responses to gain an overall understanding of participants’ perspectives. Meaning units were then identified and coded based on content relevant to motivations for surgery. These codes were subsequently condensed into thematic summaries, which were iteratively discussed and refined. Finally, condensations were synthesized into descriptive themes representing shared patterns of motivation across participants. This qualitative analysis aimed to provide a deeper understanding of participants’ perspectives, uncovering insights that may not have been captured through quantitative measures. By integrating these qualitative findings, the study sought to enrich the overall interpretation of the data, providing a more comprehensive view of the research phenomenon [15]. The qualitative analysis was conducted with continuous reflexive awareness of the researchers’ clinical and academic background (male surgeon and researcher). In line with systematic text condensation, reflexivity was treated as an integral component of the analytic process rather than as a separate methodological step. Qualitative data are presented as themes, condensed meanings, and representative quotes.

### Statistical methods

Quantitative data were presented using appropriate descriptive statistics. Continuous data were presented as means, standard deviations, medians, or interquartile ranges, as appropriate. Categorical data were presented as frequencies and percentages.

### Ethical considerations

Approval was obtained from the Danish Data Protection Agency (p-2023-14805). Demographic data were supplied by the Danish Health Data Authority (FSEID-00006834). Ethical review board approval was neither possible nor required under Danish law for a study of this nature. Informed consent was obtained from all participants.

## Results

**Fig. 1:**
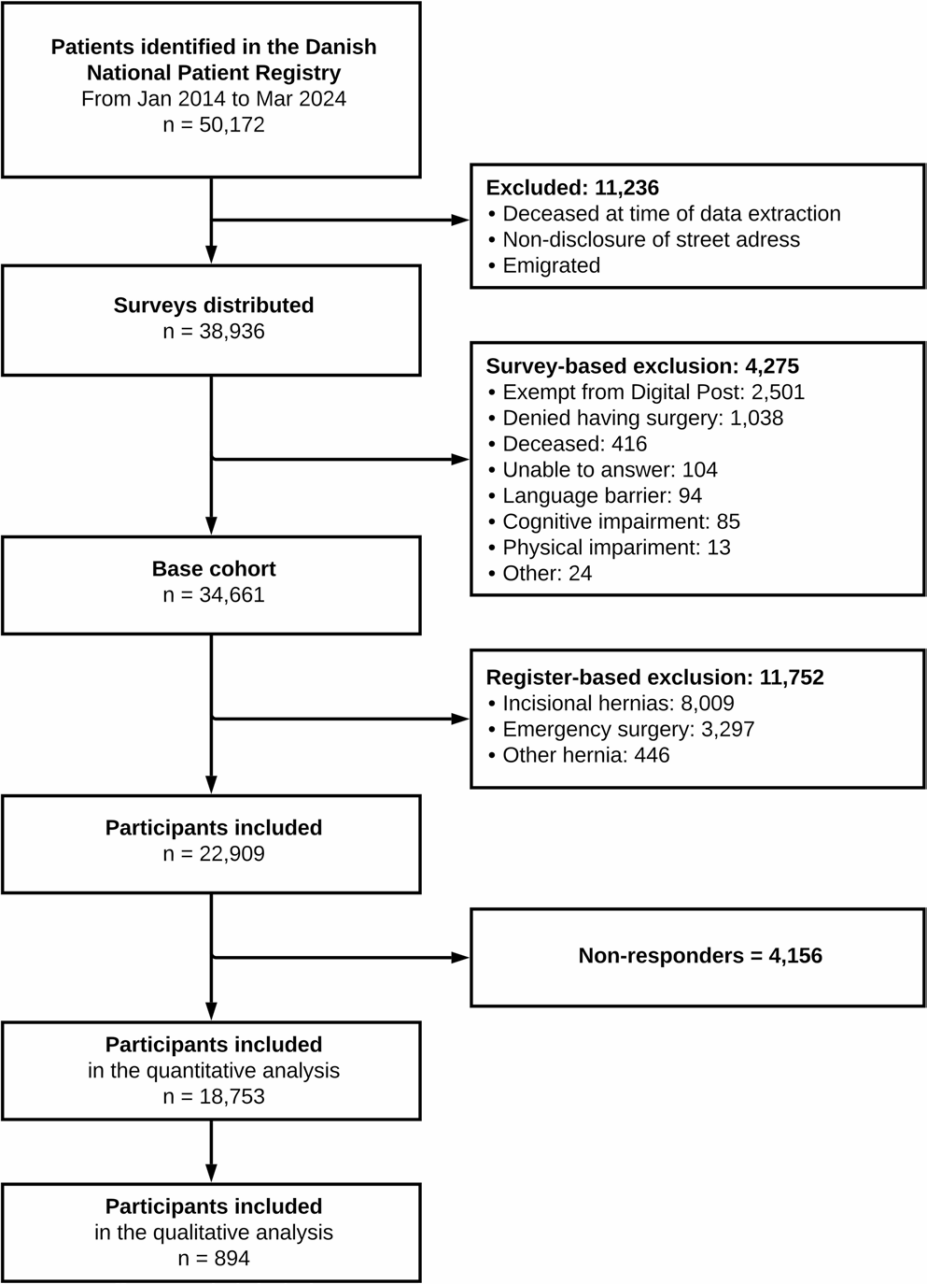
Flow-chart of participant selection

In total, 18,753 of 22,909 eligible participants responded to the survey, resulting in a response rate of 82%. Of the included participants, 33% (6,246 of 18,753) selected only one motivation for surgery, 22% (4,099 of 18,753) selected two, 20% (3,805 of 18,753) selected three, and 25% (4,603 of 18,753) selected four or more types of motivation for surgery. The participant selection process is illustrated in Fig. 1, and demographic and surgical data are shown in Table 1.

**Table 1:** Participant characteristics

Characteristic		*N* = 18,753
Women, n (%)		6,220 (33)
Age, median (IQR)		52 (42–62)
Hernia type^a^		
Umbilical hernia, n (%)		15,243 (81)
Epigastric hernia, n (%)		3,510 (19)
Defect width^b^		
< 1 cm, n (%)		3,086 (16)
1–4 cm, n (%)		13,451 (72)
> 4 cm, n (%)		474 (3)
Missing/inconsistent data^c^, (%)		1,742 (9)
Surgical approach		
Open, n (%)		15,228 (81)
Laparoscopic, n (%)		3,081 (16)
Robot-assisted, n (%)		444 (2)
Mesh/no mesh		
Mesh, n (%)		12,133 (65)
No mesh, n (%)		6,620 (35)
Follow-up time, median (IQR), months		72 (37–102)

a: If participants underwent multiple ventral hernia repairs during the study period, only their most recent operation was included

b: Maximum transverse defect width

c: Participants with no data on hernia defect size, or where data on defect size were inconsistent across sources

### Quantitative results

The most frequently selected reasons for undergoing hernia repair were pain/discomfort (63%; 11,781 of 18,753), concerns about hernia growing larger (39%; 7,262 of 18,753), and doctor’s recommendations (34%; 6,425 of 18,753). The complete distribution of participant motivation for getting surgery is listed in Table 2.

**Table 2:** Participants’ motivation for undergoing surgery

Motivation	*N* = 18,753 *n* (%)	Sex, *n* (%)		Age groups, *n* (%), years					
		Women *(n = 6,220)*	Men *(n = 12,533)*	< 30 *(n = 908)*	30–39 *(n = 2,757)*	40–49 *(n = 4,251)*	50–59 *(n = 5,114)*	60–69 *(n = 3,857)*	> 70 *(n = 1,866)*
Pain/discomfort	11,781 (63)	4,458 (72)	7,323 (58)	712 (78)	2,059 (75)	2,865 (67)	3,092 (60)	2,088 (54)	965 (52)
Fear of growth	7,262 (39)	2,102 (34)	5,160 (41)	373 (41)	1,009 (37)	1,649 (39)	2,075 (41)	1,479 (38)	677 (36)
Doctor’s recommendations	6,425 (34)	2,096 (34)	4,329 (35)	282 (31)	785 (28)	1,381 (32)	1,757 (34)	1,433 (37)	787 (42)
Cosmetic concerns	5,711 (30)	1,571 (25)	4,140 (33)	259 (29)	835 (30)	1,271 (30)	1,604 (31)	1,214 (31)	528 (28)
Exercise limitation	4,681 (25)	1,572 (25)	3,109 (25)	387 (43)	903 (33)	1,222 (29)	1,161 (23)	673 (17)	335 (18)
Work interference	4,105 (22)	951 (15)	3,154 (25)	210 (23)	599 (22)	997 (23)	1,355 (26)	747 (19)	197 (11)
Fear of incarceration	2,711 (14)	1,122 (18)	1,589 (13)	197 (22)	503 (18)	672 (16)	651 (13)	438 (11)	250 (13)
Mental impact	2,587 (14)	828 (13)	1,759 (14)	151 (17)	401 (15)	604 (14)	676 (13)	500 (13)	255 (14)
Previous incarceration	1,270 (7)	538 (9)	732 (6)	86 (9)	255 (9)	283 (7)	324 (6)	226 (6)	96 (5)
Sexual activity	1,165 (6)	270 (4)	895 (7)	87 (10)	193 (7)	307 (7)	346 (7)	163 (4)	69 (4)
Other	996 (5)	387 (6)	609 (5)	29 (3)	120 (4)	208 (5)	272 (5)	233 (6)	134 (7)

Total distribution of the participants’ responses to the following question: “Before your operation, what were the main issues that motivated you to have a hernia repair?”. It was possible for participants to select multiple response options simultaneously, and consequently, the presented percentages do not add up to 100%

In the subset of participants who exclusively selected a single reason for undergoing surgery (33%; 6,244 of 18,753), the most frequently selected were pain/discomfort (14%; 2,702 of 18,753), doctor’s recommendations (6%; 1,197 of 18,753), and cosmetic concerns (3%; 606 of 18,753). In total, 12% (2,181 of 18,753) of participants exclusively selected reasons for undergoing surgery that were classified as motivations not traditionally prioritized in surgical decision-making (i.e., cosmetic concerns, concerns about the hernia growing larger, concerns about incarceration, and mental/emotional impact).

**Fig. 2:**
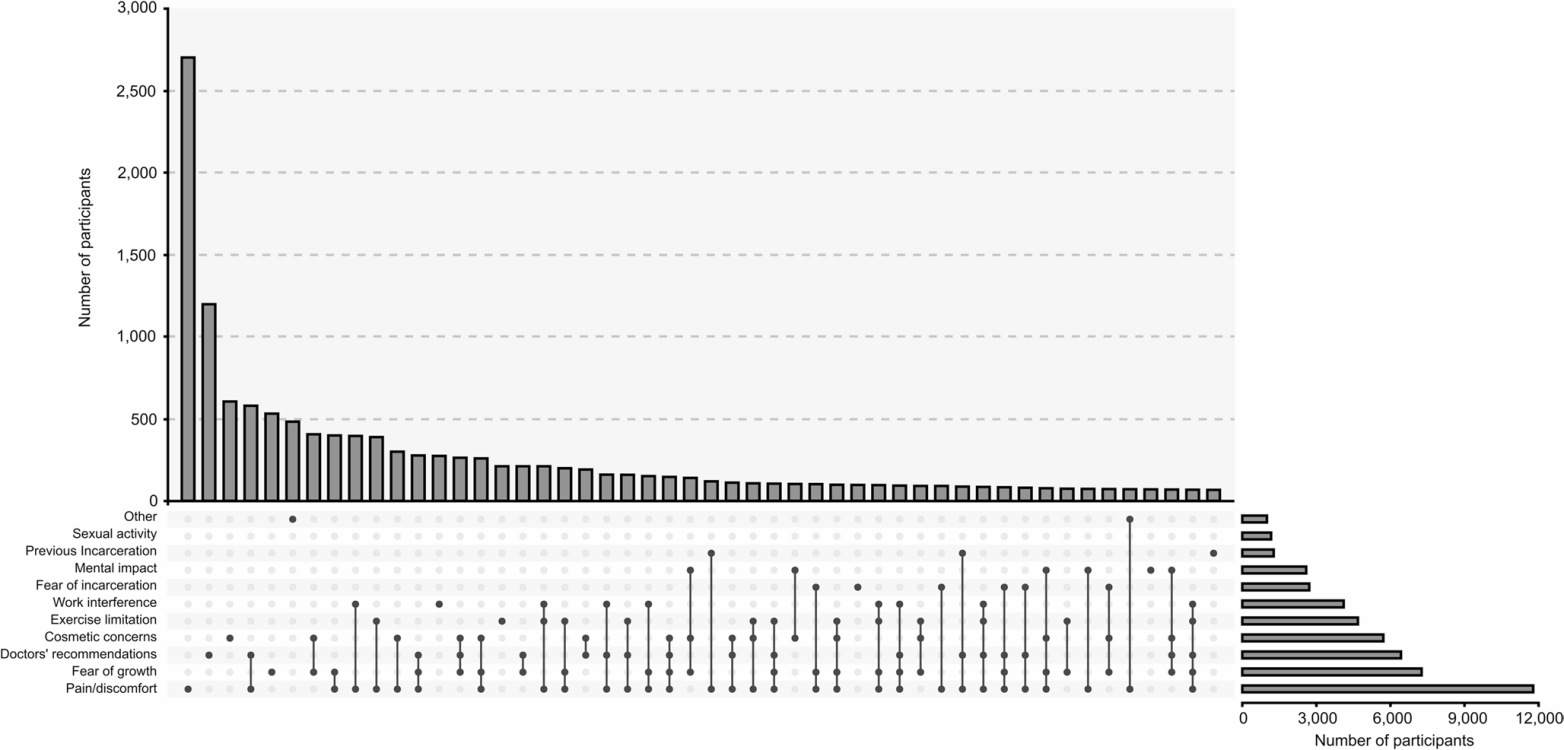
Combinations of motivations for surgery. UpSet plot [21] showing the distribution and overlap of predefined motivations for undergoing elective primary ventral hernia repair among survey respondents. Horizontal bars on the left indicate how many participants selected each motivation, while the vertical bars above represent the number of participants selecting each combination of motivations. Only the 48 most frequent combinations are shown

The three most frequently reported combinations of motivations were: pain/discomfort and doctors’ recommendations (3%; 580 of 18,753), fear of growth and cosmetic concerns (2%; 406 of 18,753), and pain/discomfort and fear of growth (2%; 399 of 18,753). The most frequent combinations of motivations are presented in Fig. 2.

**Fig. 3:**
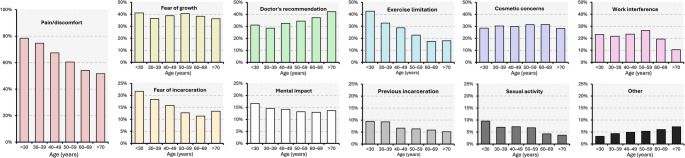
Participant motivation by age group. Participants’ replies to the question “Before your operation, what were the main issues that motivated you to have a hernia repair?”. Replies are presented as proportions by 10-year age intervals

A greater proportion of younger participants (< 50 years) (32%; 2,512 of 7,916) than older participants (≥ 50 years) (20%; 2,169 of 10,837) chose exercise-related issues as motivation for surgery. In the subgroup of participants < 30 years, an even higher proportion (43%; 387 of 908) selected exercise-related issues. Also, more younger participants (< 50 years) (71%; 5,636 of 7,916) than older participants (≥ 50 years) (57%; 6,145 of 10,837) identified pain/discomfort as a reason for undergoing surgery. In the subgroup < 30 years, this proportion was even higher (78%; 712 of 908). In general, the proportion of participants who selected pain/discomfort as motivation seemed to decline with increasing age. On the other hand, the proportion of participants who selected cosmetic concerns as motivation seemed to be quite stable at around 30% of all age groups (Fig. 3).

**Fig. 4:**
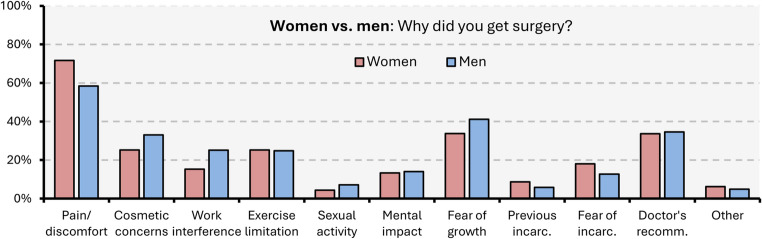
Participant motivation by sex. Participants’ replies to the question “Before your operation, what were the main issues that motivated you to have a hernia repair?”. Replies are grouped by sex

A higher proportion of women (72%; 4,458 of 6,220) than men (58%; 7,323 of 12,533) selected pain/discomfort as motivation for surgery. Conversely, more men (25%; 3,154 of 12,533) than women (15%; 951 of 6,220) identified work-related issues as motivation for getting surgery (Fig. 4). In the group of participants with a hernia defect < 1 cm, 24% (738 of 3,086) identified cosmetic concerns as motivation for surgery, while 32% (4,482 of 13,925) of participants with a hernia defect ≥ 1 cm selected cosmetic concerns as motivation for surgery.

### Qualitative results

A total of 894 participants provided free-text responses and were included in the qualitative analysis. In these free-text responses, participants described, in their own words, their reasons for undergoing elective primary ventral hernia repair. After analysis, seven overarching themes emerged, reflecting a broad spectrum of patient motivations ranging from practical convenience and medical advice to acute symptoms and cosmetic concerns. Themes, condensed meanings, and representative quotes are presented in Table 3.

**Table 3:** Themes, condensed meanings, and representative quotes

Theme		Condensed meaning	Representative quotes
1	Opportunistic or incidental repair	Surgery performed as a convenient addition to another planned procedure. Decision often surgeon-initiated.	“*The surgeon repaired it while I was already in for another operation.*” “*He said we might as well fix it now.*”
2	Acute worsening and fear of complications	Acute pain or swelling triggered anxiety about incarceration or bowel obstruction. Surgery perceived as urgent or preventive.	“*It became painful and I couldn’t push it back.*” “*I was afraid it could get stuck.*”
3	Physical discomfort and everyday limitations	Ongoing irritation or functional restriction in work or exercise. Surgery aimed to restore comfort and activity.	“*It hurt when I moved.*” “*It rubbed against my pants and was constantly annoying.*”
4	Doctor’s recommendation and professional authority	Operation primarily based on doctor’s advice. Patients trusted professional judgment over self-motivation.	“*My doctor said it was better to do it now.*” “*I left the decision up to the surgeon.*”
5	Cosmetic concerns and body awareness	Motivation related to appearance or embarrassment. Desire to restore normal bodily appearance.	“*It looked ugly.*” “*I wanted my navel to look normal again.*”
6	Recurrent hernias and frustration	Previous failed repairs created frustration and desire for a final, lasting solution.	“*It was my second operation.*” “*I wanted it done properly this time.*”
7	Unaware or unintentional decision	Surgery followed incidental discovery of hernia. Motivation was largely absent or unrecognized.	“*I didn’t know I had a hernia.*” “*It was fixed without me asking.*”

Theme 1: Opportunistic or incidental repair. This theme comprised participants whose hernia was repaired during another planned procedure, typically at the surgeon’s initiative. For these individuals, the decision was pragmatic and opportunistic rather than actively pursued. Theme 2: Acute worsening and fear of complications. This theme included participants who experienced sudden pain, swelling, or difficulty reducing the hernia. Acute symptom progression or concerns about incarceration prompted a sense of urgency for repair. Theme 3: Physical discomfort and everyday limitations. The theme encompassed participants who described persistent irritation, pain, or functional impairment during work or exercise. Repair was sought to relieve discomfort and restore physical capability. Theme 4: Doctor’s recommendation and professional authority. The decision to proceed was primarily guided by medical advice. Participants expressed trust in the doctor’s judgment and perceived the operation as a professionally led decision. Theme 5: Cosmetic concerns and body awareness. This theme represented participants motivated by aesthetic discomfort or body-image considerations. The hernia’s visible protrusion caused embarrassment or dissatisfaction with appearance. Theme 6: Recurrent hernias and frustration. This theme included individuals who had previously undergone hernia surgery. Reoperation was motivated by frustration with recurrence and the wish for a lasting solution. Theme 7: Unaware or unintentional decision. This reflected participants who had not actively chosen surgery. For these, the hernia was discovered incidentally, and the repair occurred at the surgeon’s discretion.

In general, the qualitative analysis of the free-text responses did not reveal any fundamentally different motivations than the prespecified response options and the quantitative data, as there was a large overlap in the types of motivation for surgery that emerged from the qualitative analysis and the prespecified types of motivation. This may suggest that the prespecified types of motivation are an appropriate representation of patients’ preoperative decision-making and motivation for undergoing elective primary ventral hernia repair.

## Discussion

This nationwide mixed-methods study explored patients’ motivations for undergoing elective repair of a primary ventral hernia. About one in eight respondents underwent hernia repair for reasons that are not traditionally prioritized in surgical decision-making, such as emotional impact and cosmetic concerns. Overall, pain and discomfort were the most frequently reported reasons for surgery, followed by concerns about hernia progression and the doctor’s recommendations. The proportion of participants motivated by pain/discomfort seemed to decline with increasing age, but the proportion of participants motivated by cosmetic concerns seemed to remain stable regardless of age. The qualitative analysis supported the quantitative findings and provided additional context, indicating that most patients were motivated by symptom burden or professional advice rather than cosmetic or emotional concerns. Doctor’s recommendation emerged as a prominent motivation, underscoring the influence of professional authority in surgical decision-making. For many patients, medical advice may provide reassurance and serve as an important guide in situations where indications are not always clear-cut. This also underscores the role of shared decision-making, where clinical expertise and patient preferences must be aligned. In a healthcare system such as Denmark’s, characterized by high levels of trust in public healthcare, medical advice may strongly shape patients’ decisions, which should be considered when interpreting these findings in other healthcare settings.

### Strengths and limitations

The principal strengths of this study are its large, nationwide design, linkage to validated clinical registries, and use of a mixed-methods approach integrating structured and open-ended data. This combination increased both the external validity and interpretability of the findings.

Limitations include the risk of recall bias, as motivations were self-reported and may have been influenced by postoperative experiences and outcomes. Motivations were assessed retrospectively, and therefore the responses may reflect retrospective narratives rather than the decision-making at the actual time of surgery. This is an inherent limitation of a retrospective study based on patient-reported data with a long follow-up time. Furthermore, the prespecified types of motivation were defined exclusively by the author group based on personal and clinical experience without direct patient involvement, which presents a risk of framing bias and a risk of omitting relevant input. To minimize these biases, we implemented the ”Other” response option and the free-text option, which enabled participants to reply using their own words. The influence of non-response bias cannot be excluded, as participation was voluntary. We did not account for concomitant surgery, such as diastasis recti repair, which may have introduced unmeasured confounding. The exclusion of individuals with limited digital access or language proficiency may have affected representativeness. In the qualitative analysis, all interpretations were naturally influenced by the researchers’ perspectives, but we aimed to maintain reflexive awareness throughout the analytic process. Nonetheless, results from the qualitative analysis were inevitably influenced to some degree by our own preconceptions, which included the prespecified types of motivation from the quantitative analysis. Finally, some free-text responses were short and lacked detail, which may have limited the depth of qualitative interpretation.

### Clinical implications

The findings of this study underline the importance of addressing patient motivation and expectations in the preoperative evaluation of ventral hernias. As in other surgical fields, patients’ decisions are influenced not only by symptoms but also by perceptions of disease severity, body image, and professional advice [16, 17]. Although most patients in the present study sought surgery for specific symptom relief, a considerable proportion was motivated by concerns about hernia growth or complications, which are factors that generally do not align with clinical indications for surgery. Similar discrepancies between perceived and actual surgical necessity have been observed in studies exploring patient knowledge and beliefs about hernia surgery [18].

Given the increasing availability of online information, patients’ understanding of hernia-related risks and treatment options is shaped by diverse, sometimes inaccurate sources [19]. Surgeons therefore play a critical role in clarifying the natural history of small or minimally symptomatic hernias, explaining when surgery is and is not indicated. Transparent communication about expected benefits, recovery, and potential complications is essential to ensure realistic expectations and avoid postoperative dissatisfaction. As shown in recent work, unfulfilled expectations are a key predictor of decisional regret following ventral hernia repair [20]. Integrating patient motivations into shared decision-making may therefore enhance satisfaction, improve surgical outcomes, and reduce regret.

## Conclusion

This study found that most patients undergo elective repair of a primary ventral hernia due to pain or discomfort, concerns about their hernia growing larger, and because of their doctor’s recommendations. Patients’ sex and age affected their motivation for undergoing surgery. Furthermore, 12% of respondents were motivated exclusively by cosmetic or emotional factors, which are indications not traditionally prioritized in surgical decision-making.

## Data Availability

Due to Danish legislation, supporting data are not available.
